# Non-surgical Management of Neonatal Esophageal Perforation: A Rare Case

**DOI:** 10.7759/cureus.77289

**Published:** 2025-01-11

**Authors:** Muhammad Mudasir Saleem, Mishal Pervaiz, Ismail Mazhar, Uswah Shoaib, Muhammad Osama, Amaan Ahmad, Sehar Khauteja Khan, Muhammad Ibrahim Tahir, Khawaja Haider Sami, Maida Muazzam

**Affiliations:** 1 Pediatric Surgery, Combined Military Hospital (CMH), Lahore, PAK; 2 Anesthesiology, Combined Military Hospital (CMH), Lahore, PAK; 3 Internal Medicine, Combined Military Hospital (CMH) Lahore Medical College, Institute of Dentistry, Lahore, PAK; 4 Pediatric Surgery, Combined Military Hospital (CMH) Lahore Medical College, Institute of Dentistry, Lahore, PAK; 5 Internal Medicine, Lahore Medical and Dental College, Lahore, PAK; 6 General Surgery, Shifa Tameer-E-Millat University Shifa College of Medicine, Islamabad, PAK

**Keywords:** iatrogenic esophageal perforation (iep), neonatal intensive care unit (nicu), neonates, orogastric tube, respiratory distress

## Abstract

Iatrogenic esophageal perforation in neonates, though rare, is a serious condition with high mortality. It is almost exclusively secondary to invasive instrumentation complications in intensive care settings. Forceful or repeated orogastric (OG) or nasogastric (NG) tube insertion, vigorous suctioning to clear the airway, and trauma during airway intubation are the leading causes. Being rare in occurrence, a high index of suspicion should be maintained for early diagnosis and prompt treatment to prevent mortality. We present a similar case in a 2.6 kg male neonate who developed this complication from OG tube placement in the neonatal intensive care unit during the treatment of respiratory distress and parapneumonic effusion.

## Introduction

Iatrogenic esophageal perforation (IEP) is a rare but serious condition in neonates, particularly those who are premature or have low birth weight, with a reported mortality rate of around 30% [1]. Neonates are prone to IEP due to fragile esophageal tissues and underdeveloped musculature, with common causes including endotracheal intubation, nasogastric (NG) tube placement, or gastrointestinal suctioning [2]. Rarely, spontaneous perforations may occur from increased intraesophageal pressure during severe crying or vomiting [3]. Most perforations occur at the pharyngoesophageal junction, with risk factors including weaker pharyngeal muscles and neck hyperextension during tube insertion [4]. The lack of a protective serous layer increases vulnerability to complications such as mediastinitis, empyema, and sepsis, contributing to high mortality [5]. Presentation often includes respiratory distress, subcutaneous emphysema, and bloody aspirate after difficult intubation or tube placement [6]. Imaging typically reveals pneumothorax, pneumomediastinum, or a malpositioned tube [7]. Early recognition and management, including cessation of feeding, antibiotics, and monitoring, are critical for improving outcomes [8]. Our case was notable for the absence of typical risk factors. It was diagnosed via a feed and identified in a chest drain placed for parapneumonic effusion.

## Case presentation

**Figure 1:**
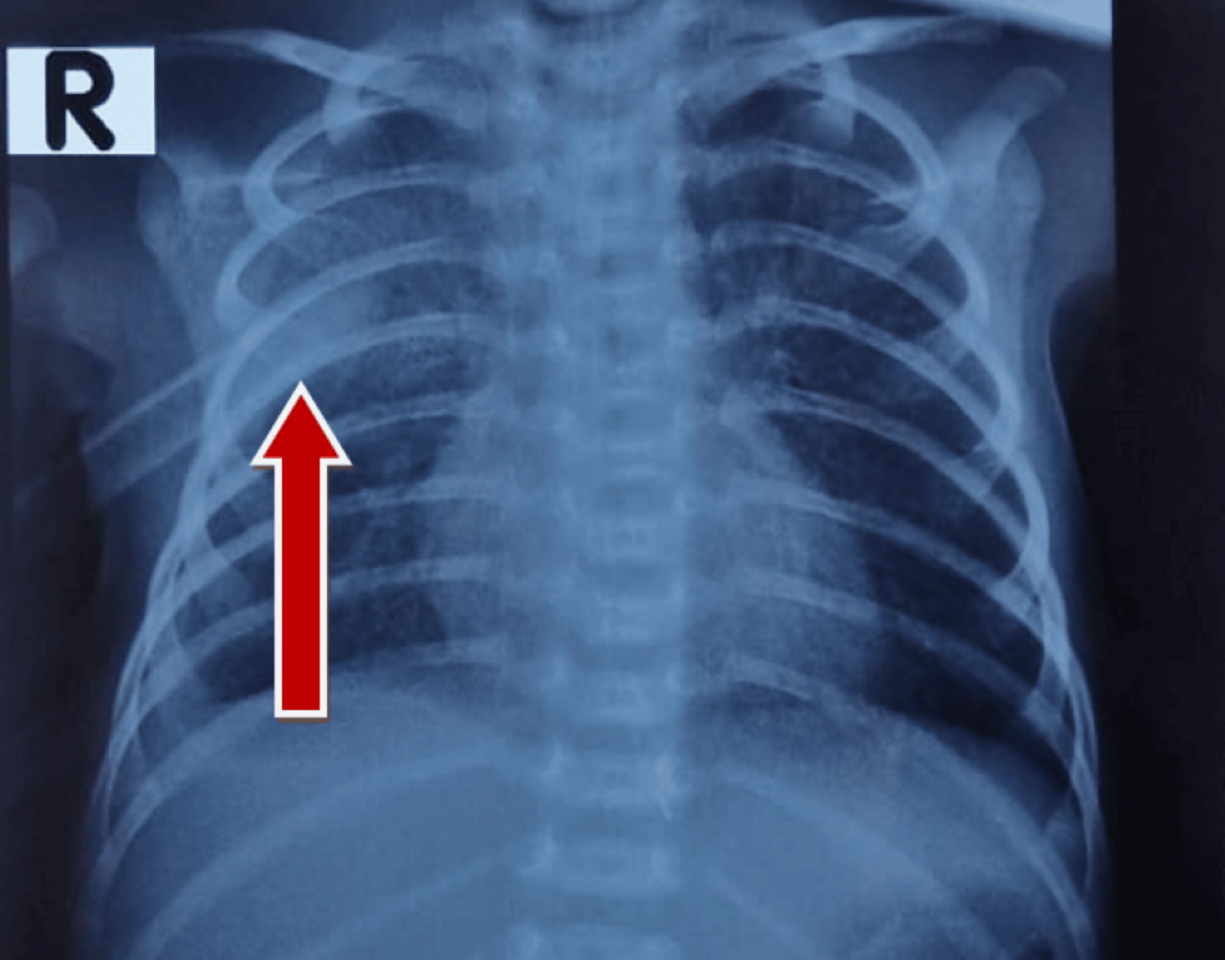
Initial chest X-ray with chest tube on right side (red arrow)

A 2.6 kg male neonate was delivered via lower segment cesarean section at term after an uneventful pregnancy and was admitted to the neonatal intensive care unit on the sixth day of neonatal life with respiratory distress and early-onset neonatal sepsis. The infant was placed on continuous positive airway pressure-assisted ventilation to maintain oxygen saturation due to increasing respiratory distress. A chest X-ray revealed parapneumonic effusion in the right chest, for which a right-sided chest tube was placed by the pediatric surgery team. The tube yielded serous fluid positive for neutrophils (Figure 1).

**Figure 2:**
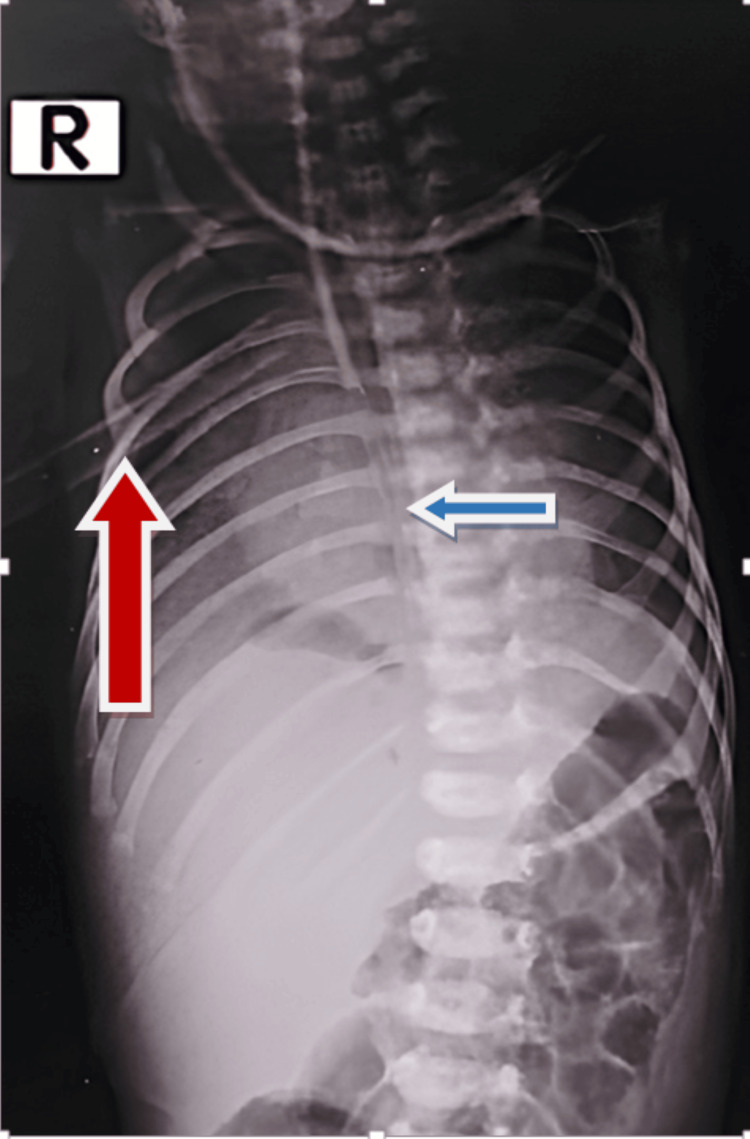
Misplaced orogastric tube in right chest cavity marked with blue arrow (chest tube marked with red arrow)

Broad-spectrum antibiotics were initiated. A follow-up chest X-ray revealed resolution of the effusion with improvement in respiratory distress. The orogastric (OG) tube, which had been initially placed, was accidentally removed the same evening. The on-duty resident reinserted the OG tube, and proper positioning was confirmed by insufflating air and auscultation. The following day, milk feed was administered, and the duty staff noted milky fluid in the chest drain output. A chest X-ray revealed free air in the mediastinum and a malpositioned OG tube tip in the right chest cavity (Figure 2).

**Figure 3:**
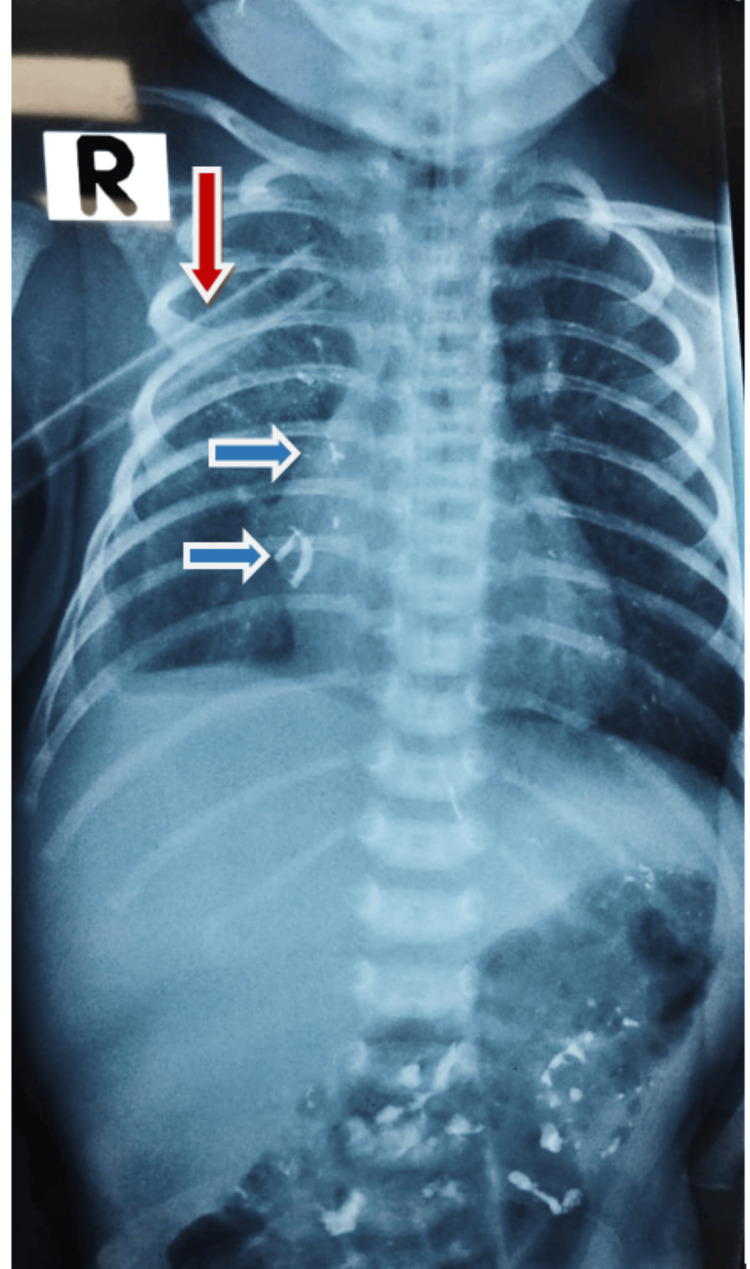
Diluted barium esophagogram with contained leak in right chest cavity marked with blue arrows ( chest tube marked with red arrow)

An urgent upper gastrointestinal contrast study with diluted barium was ordered to confirm the diagnosis and quantify the leak. The imaging confirmed that the OG tube was misplaced in the right chest cavity instead of the stomach, with minimal contrast extravasation indicating an esophageal perforation (Figure 3)

**Figure 4:**
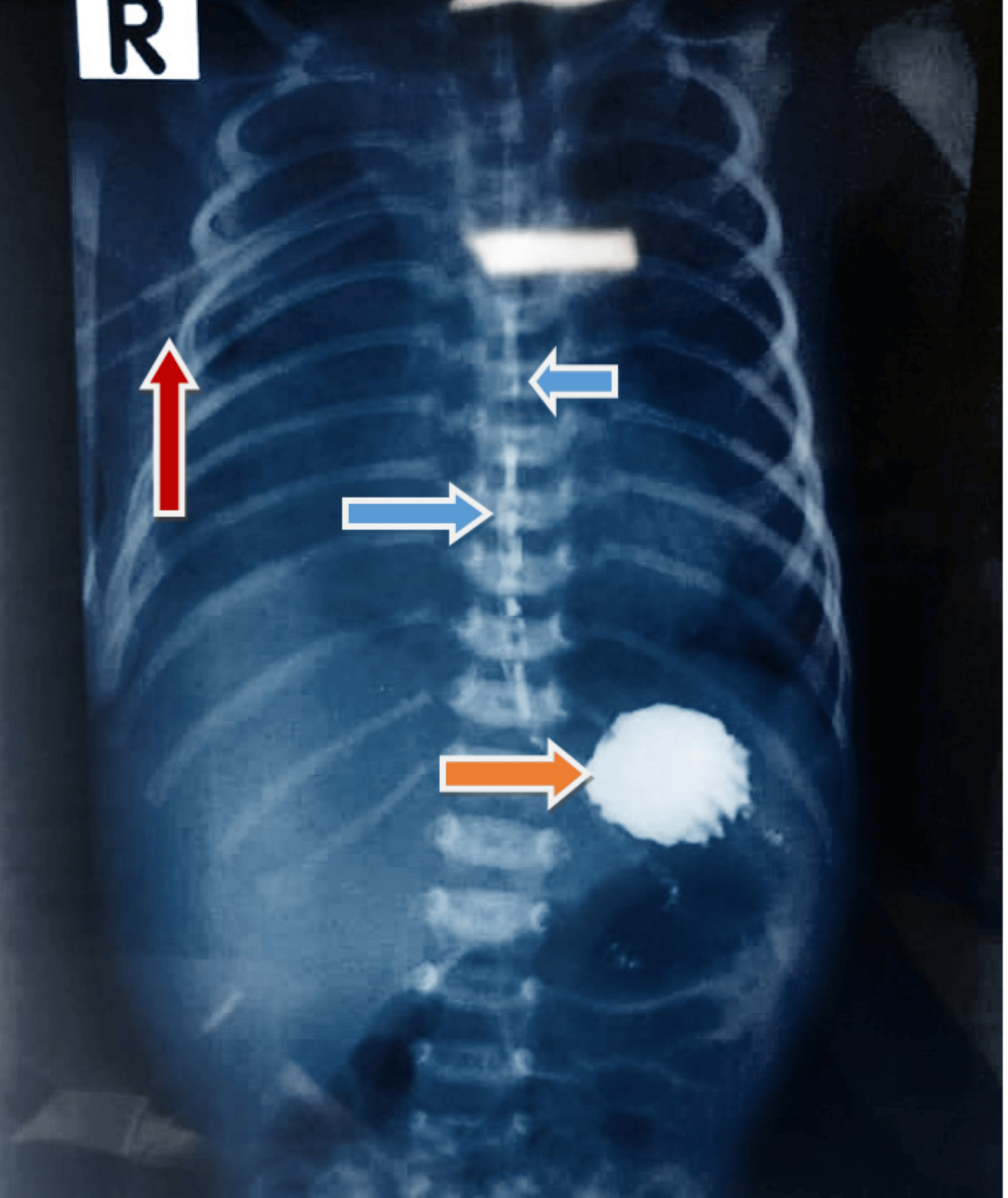
Repeat upper gastrointestinal follow through with no contrast extravasation in chest marked with blue arrow. Contrast in stomach is marked with orange arrow and chest tube is marked with red arrow

The pediatric surgery team was consulted, and the parents were counseled in detail. A multidisciplinary team discussion established a conservative management plan, given the neonate's hemodynamic stability and the contained leak, with an already placed chest thoracostomy. The baby was kept nil per oral, total parenteral nutrition was started, and antibiotics were escalated to more targeted coverage. The misplaced OG tube was gently removed. Following conservative management, a repeat upper gastrointestinal follow-through on the 14th day showed no extravasation and contrast-free flow into the stomach, with proper delineation of esophageal integrity (Figure 4). Feeding was gradually initiated, and the chest drain was removed. The neonate was discharged on the 25th day with full oral feeds. Serial follow-ups revealed a thriving baby with no swallowing or respiratory issues.

## Discussion

Esophageal perforation is an extremely rare emergency, with an estimated incidence of approximately 0.006% [9]. Warden first reported neonatal IEP in 1961 [4]. In neonates, esophageal perforation is exclusively iatrogenic and secondary to instrumentation in the intensive care unit. OG/NG tube insertion is the leading reported cause in the literature, as in our report [10]. While low birth weight and prematurity are considered the main risk factors, a review of the literature showed that IEP has also been reported in cases with normal gestation and birth weight, though these are even rarer. Our case involved a full-term neonate with a birth weight of 2.6 kg, placing it in the rarer category of IEP presentation.

The usual presentation includes nonspecific symptoms such as blood-tinged aspirate, respiratory distress, and sepsis-like signs, which can overlap with other conditions. Differential diagnoses for such presentations include spontaneous pneumothorax, congenital diaphragmatic hernia, esophageal atresia or fistula, mediastinitis due to infectious causes, tracheoesophageal fistula, and aspiration pneumonia. Imaging is critical in distinguishing IEP from these conditions.

Key diagnostic indicators for IEP include difficulty passing an OG/NG tube and abnormal findings on chest X-rays, such as subcutaneous emphysema, pneumomediastinum, pneumothorax, pleural effusion, or atelectasis [11]. Additional workup, including a computerized tomography (CT) chest scan or esophagram with diluted barium contrast, can confirm the diagnosis in doubtful cases [12]. Another utility of the barium esophagogram is quantifying the leak, as was done in our case before proceeding with non-operative management.

It is mandatory to confirm the position of the OG/NG tube radiologically after each insertion and not simply rely on clinical confirmation by insufflating air and auscultation in the epigastrium, which can give a false sense of tube position confirmation in the stomach. Our patient missed this important step. Similarly, starting feeds without this confirmation can be disastrous. In our case, the diagnosis was made by identifying milk feed in the already placed chest tube, which could have easily been missed if the chest tube had not been there, potentially delaying the diagnosis with negative outcomes. Non-operative management is the preferred treatment for IEP in neonates, considering hemodynamic stability and a contained leak. This approach includes keeping the neonate nil per oral, providing intravenous fluids, parenteral nutrition, broad-spectrum antibiotics, and avoiding OG/NG tube insertion with minimal oropharyngeal suctioning to prevent further injury [2].

Sticco et al. reported a case in a premature neonate where IEP was diagnosed by a blood-stained oropharyngeal aspirate following OG tube placement. The condition was successfully managed non-operatively with a positive outcome due to the patient’s hemodynamic stability [13]. Borries et al. reported a case of IEP in a 23-week, low-birth-weight neonate who developed this complication after OG tube placement. A water-soluble contrast esophagogram confirmed the diagnosis, and the condition was managed conservatively with broad-spectrum pharmacotherapy [14]. Ben Aoun et al. reported another case of IEP in a male neonate that was misdiagnosed as esophageal atresia after repeated failed attempts to insert an NG tube. The perforation was diagnosed peri-operatively and repaired primarily, highlighting the importance of adequate workup to avoid unnecessary surgical intervention [15]. Contrary to the operative management of esophageal perforation in adults, non-operative conservative management has evolved as the gold standard for the treatment of IEP in children, with promising outcomes [16].

## Conclusions

Neonatal IEP is a rare surgical emergency that is challenging due to limited clinical exposure and a lack of evidence-based guidelines. Early recognition of nonspecific symptoms such as respiratory distress, blood-tinged aspirate, or sepsis-like signs, combined with imaging like chest X-rays and esophagrams, is critical for accurate diagnosis. Preventative measures include using appropriately sized and softened tubes, confirming proper positioning through imaging or pH testing before feeding, and adhering to standardized insertion protocols to minimize trauma. Management depends on severity: stable cases may respond to cessation of feeds, antibiotics, and monitoring, while severe cases may require surgical repair. Improving preventative practices, early diagnosis, and timely intervention can significantly improve outcomes for neonatal IEP.
